# *European Journal of Heart Failure* consensus statement. Heart failure pharmacotherapy for patients with heart failure with reduced ejection fraction and concomitant atrial fibrillation: Review of evidence and call to action

**DOI:** 10.1002/ejhf.70069

**Published:** 2025-10-22

**Authors:** Mark Luedde, Stefan Agewall, Giuseppe Ambrosio, Antoni Bayes‐Genis, Claudio Borghi, Elisabetta Cerbai, Gheorghe A. Dan, Heinz Drexel, Péter Ferdinandy, Erik Lerkevang Grove, Juan Carlos Kaski, Roland Klingenberg, Joao Morais, William Parker, Mark C. Petrie, Bianca Rocca, Anne Grete Semb, Michele Senni, Christian Sohns, Patrick Sulzgruber, Juan Tamargo, Marco Metra, Michael Böhm, Dobromir Dobrev, Samuel Sossalla

**Affiliations:** ^1^ Medical Clinic I, Cardiology and Angiology, Justus‐Liebig‐University Giessen Germany; ^2^ Cardiologicum Bremerhaven, Sanecum Group Bremerhaven Germany; ^3^ Department of Clinical Sciences Danderyd Hospital, Karolinska Institute Stockholm Sweden; ^4^ Department of Medicine and CERICLET University of Perugia School of Medicine Perugia Italy; ^5^ Institute for Cardiovascular Research ‐INRC Perugia Italy; ^6^ Heart Institute, Hospital Universitari Germans Trias I Pujol, CIBERCV Badalona Spain; ^7^ Department of Cardiovascular Medicine University of Bologna‐IRCCS AOU S. Orsola Bologna Italy; ^8^ Department Neuroscience Section of Pharmacology and Toxicology, University of Florence Florence Italy; ^9^ Carol Davila University of Medicine and Pharmacy, Academy of Romanian Scientists Bucharest Romania; ^10^ Vorarlberg Institute for Vascular Investigation & Treatment (VIVIT) Feldkirch Austria; ^11^ Department of Pharmacology and Pharmacotherapy Semmelweis University Budapest Hungary; ^12^ Pharmahungary Group Szeged Hungary; ^13^ Center for Pharmacology and Drug Research & Development, Semmelweis University Budapest Hungary; ^14^ Department of Cardiology Aarhus University Hospital Aarhus Denmark; ^15^ Department of Clinical Medicine Faculty of Health, Aarhus University Aarhus Denmark; ^16^ Cardiovascular and Cell Sciences Research Institute at St George's, University of London London UK; ^17^ Medical Clinic I, Cardiology and Angiology, Justus‐Liebig‐University, Giessen and Department of Cardiology, Kerckhoff‐Clinic/DZHK Bad Nauheim Germany; ^18^ Krankenhaus Nordwest, Stiftung Hospital zum Heiligen Geist Frankfurt Germany; ^19^ ciTechCare ‐ Center for Innovative Care and Health Technology. Polytechnic University of Leiria Leiria Portugal; ^20^ Cardiovascular Research Unit, University of Sheffield Sheffield UK; ^21^ School of Cardiovascular and Medical Sciences, University of Glasgow Glasgow UK; ^22^ Department of Medicine and Surgery, LUM University Casamassima Italy; ^23^ Preventive Cardio‐Rheuma clinic, Division of Research and Innovation, REMEDY Centre, Diakonhjemmet Hospital Oslo Norway; ^24^ University of Milano‐Bicocca, Cardiovascular Department, Papa Giovanni XXIII Hospital Bergamo Italy; ^25^ Department of Electrophysiology, Herz‐ und Diabeteszentrum NRW, Ruhr‐Universität Bochum Bad Oeynhausen Germany; ^26^ Department of Medicine Division of Cardiology, Medical University of Vienna Vienna Austria; ^27^ Department of Pharmacology and Toxicology School of Medicine, Universidad Complutense, Instituto de Investigación Sanitaria Gregorio Marañón Madrid Spain; ^28^ Cardiology, ASST Spedali Civili, Department of Medical and Surgical Specialties, Radiological Sciences, and Public Health University of Brescia Brescia Italy; ^29^ Klinik für Innere Medizin III, HOMICAREM (HOMburg Institute for CArdioREnalMetabolic Medicine), Universitätsklinikum des Saarlandes, Saarland University Homburg/Saar Germany; ^30^ Institute of Pharmacology, West‐German Heart and Vascular Center, University Duisburg‐Essen Essen Germany; ^31^ Department of Medicine Montreal Heart Institute and Université de Montréal Montréal QC Canada; ^32^ Department of Integrative Physiology Baylor College of Medicine Houston TX USA; ^33^ Excellence Cluster Cardiopulmonary Insitute (CPI) Klinikstraße 33 Giessen Germany

**Keywords:** Heart failure, Atrial fibrillation, Cardiovascular pharmacotherapy

## Abstract

Heart failure (HF) and atrial fibrillation (AF) are major global health challenges with rising prevalence and significant morbidity, mortality, and healthcare burden. Despite advances in HF management, AF remains a critical comorbidity that worsens outcomes and requires ad hoc treatment strategies, increasing the risk of non‐adherence and side effects. While rhythm control strategies in AF have gained attention for their prognostic benefits in HF, the pharmacological treatment of HF in patients with AF, including the benefit of rhythm versus rate control, remains underexplored. The relationship between HF and AF lacks sufficient evidence and targeted research to assess the optimal treatment strategies. This narrative review critically examines current HF pharmacotherapy in the context of AF, focusing on the four cornerstone treatments and modifiers of prognosis for HF with reduced ejection fraction: beta‐blockers, angiotensin‐converting enzyme inhibitors/angiotensin receptor blockers/sacubitril‐valsartan, aldosterone antagonists, and sodium–glucose co‐transporter 2 inhibitors. Although these therapies are well‐established in HF patients, their efficacy in patients with concomitant AF requires further prospective investigation. The unique challenges posed by AF, including arrhythmia‐induced remodelling and cardiomyopathy, necessitate a more individually tailored treatment. We also highlight critical knowledge gaps and the need for dedicated clinical trials specifically assessing HF therapies in AF subgroups, such as paroxysmal, long‐standing persistent and permanent AF, and the benefit of heart rate and rhythm control strategies. The future of precision medicine in HF‐AF management lies in bridging these evidence gaps through targeted research and interdisciplinary collaboration.

## Introduction

Whereas the incidence of certain non‐communicable diseases such as coronary heart disease has fallen slightly in the Western world in recent decades,^1^ two other common cardiological conditions are still on the rise: heart failure (HF) and atrial fibrillation (AF). Both AF^2^ and HF^3^ have been described as the epidemic of the 21st century. An estimated 56.2 million people worldwide suffer from HF.^4^ Although the incidence of HF has stabilized or even declined over the past decade in high‐income countries, the prevalence continues to increase due to a number of factors, including ageing, increase in cardiovascular risk factors, more effective diagnostics, and improved survival rates (also as a result of new HF therapies^4^). Between 2010 and 2019, the global prevalence of AF has risen markedly from 33.5 million to 59 million individuals.^2^ The incidence rate of AF is 10‐fold higher in patients with HF^5^: up to 50% of HF patients suffer from AF, with the prevalence increasing with HF severity. Conversely, 30% of AF patients suffer from HF.^6^, ^7^

Both entities are associated with significant morbidity and mortality and place a heavy burden on healthcare systems. AF is responsible for 10–25% of all ischaemic strokes^8^, ^9^ and is associated with increased rates of dementia^10^ and even epilepsy.^11^ Likewise, despite established therapies (as outlined below), the mortality rate for HF remains high, with a 5‐year mortality rate of 50–70%.^4^, ^12^ Not only is mortality high, which is associated with a high rate of hospitalization and a severely impaired quality of life, but those affected are also at risk of other comorbidities, such as renal failure^13^ or increased rates of depression and dementia.^14^ Comorbidities in particular influence the course of HF, including symptoms and prognosis.^15^

**Figure 1:**
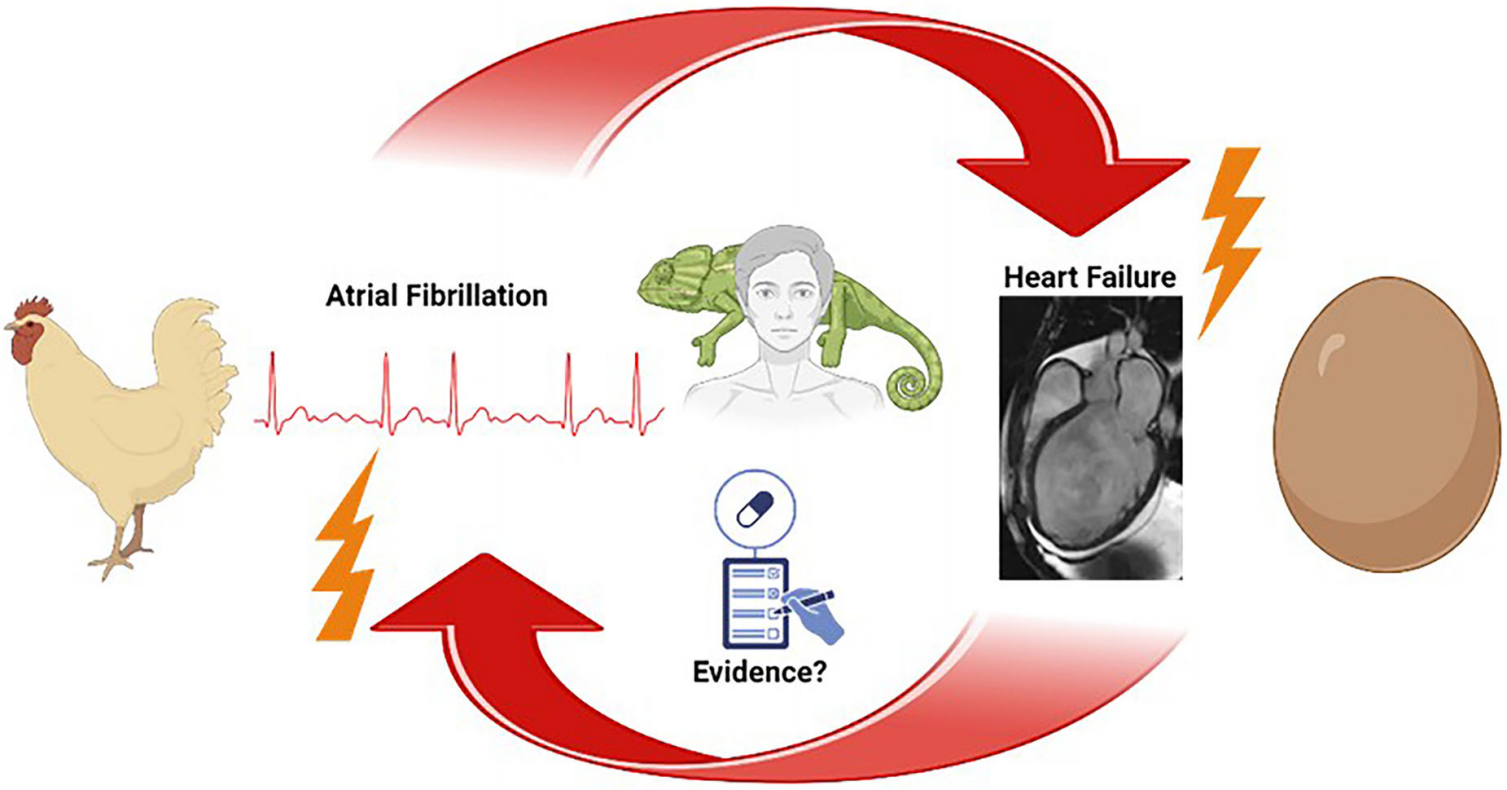
The bidirectional relationship between heart failure (HF) and atrial fibrillation (AF). The illustration highlights the complex interplay between HF and AF, where each condition can induce the other. At the time of initial patient presentation, it is often unclear whether HF or AF developed first or if both emerged simultaneously. This diagnostic and therapeutic challenge is symbolized by the chameleon, representing the elusive and variable nature of the disease. Given this complexity, pharmacological treatment remains insufficiently studied and lacks strong evidence‐based guidance.

Most importantly, AF is one of the most frequent and serious comorbidities of HF,^16^ and the two entities are mutually dependent.^17^, ^18^ In the Swedish Heart Failure Registry, the prevalence of, association with, and prognostic impact of AF in HF across all HF phenotypes were investigated. The prevalence of AF in these HF patients was remarkably high: 65% in HF with preserved ejection fraction (HFpEF), 60% in HF with mildly reduced ejection fraction (HFmrEF), and 53% in HF with reduced ejection fraction (HFrEF).^19^ Lower values were found in other registries that likely had more selected study populations. In the European Society of Cardiology (ESC) HF Long‐Term registry, the prevalence of AF was 20% in patients with HFrEF, 22% in those with HFmrEF, and 32% in those with HFpEF.^20^ New‐onset AF seems to worsen the prognosis of HF patients while in long‐standing persistent AF, a clear impact on prognosis was not clearly detectable in several studies after adjustment.^21^, ^22^, ^23^, ^24^, ^25^, ^26^ The risk of developing HF in the presence of AF is three times higher.^27^ Conversely, AF can also be a result of HF, suggesting a complex interplay between the two entities^21^, ^28^, ^29^ (*Figure* *1*). In addition, arrhythmia‐induced cardiomyopathy (AiCM) can occur with or without pre‐existing HF.^30^, ^31^, ^32^ Tachycardia does not necessarily have to be present (tachymyopathy); irregularity in AF can be sufficient to cause left ventricular systolic dysfunction.^33^, ^34^, ^35^, ^36^ In a recent prospective trial, we observed a prevalence of AiCM as high as 82% in the specific but relevant patient cohort with otherwise unexplained left ventricular systolic dysfunction and concomitant tachyarrhythmia, suggesting that this condition may be underdiagnosed in clinical practice.^32^ This complex interaction between the two conditions with regard to their epidemiological clustering underscores its clinical and pathogenetic importance.

Recent cardiovascular research has primarily focused on the comorbidities of type 2 diabetes, obesity, and chronic kidney disease in the context of HF, but the high prevalence of concomitant AF and HF underscores the growing global health burden of these entities and highlights the urgent need for a more individualized and evidence‐based treatment. While rhythm control therapy for AF in HF is gaining increasing importance—with growing evidence of its prognostic benefits and positive effects on left ventricular function^32^, ^33^, ^37^—pharmacological treatment of HF in the context of concomitant AF remains poorly explored. In this context, the historically organ‐centred perspective on HF has increasingly shifted towards a focus on comorbidities, emphasizing the need for development of novel therapies and more personalized treatment approaches that take into account common comorbidities.^38^ There is a need for more research targeting the mechanisms of HF‐associated AF, but some promising novel mechanisms for potential drug targets have been explored.^39^

Here we provide a critical review of pharmacotherapeutic strategies and the supporting evidence in patients with HF and concomitant AF, and we highlight current gaps in knowledge evidence and management. The first part of the document examines the evidence for the main HF therapies in this AF subgroup. Particular emphasis is placed on the data supporting the four pillars of drug therapy for HFrEF determined in the 2021 ESC guidelines^15^ and 2023 update^40^: (1) beta‐blockers, (2) angiotensin‐converting enzyme (ACE) inhibitors/angiotensin receptor blockers (ARBs)/sacubitril‐valsartan, (3) aldosterone antagonists, and (4) sodium–glucose co‐transporter 2 (SGLT2) inhibitors. Since therapeutic evidence in patients with HFpEF is not as clear as in those with HFrEF, we will primarily focus on patients with HFrEF. The second part of this review highlights where evidence needs to be generated, i.e. where and how scientific and clinical actions are needed to improve personalized medicine for these patients.

## Heart failure drug therapy in patients with atrial fibrillation

### Beta‐blockers

Beta‐blockers are among the longest‐established components of HF drug therapy, with proven prognostic benefits and extensive clinical experience.^15^ They belong to the four classes of drugs that improve left ventricular remodelling^41^ in HF and have a positive effect on the symptoms of HF^42^ as well as on morbidity and mortality.^43^, ^44^, ^45^, ^46^ Beta‐blockers have various effects on HF, including restoration of the downregulated beta‐adrenergic receptors and protection from the untoward effects of heightened cardiac sympathetic drive.^47^ The negative chronotropic effect may also be desirable at least in some patients, namely those in sinus rhythm (SR)^15^; this evidence is considered in the ESC IIa recommendation to further reduce the heart rate in these patients with HFrEF, and at heart rate >70 bpm by administering the I_f_ channel blocker ivabradine.^15^, ^48^ There is evidence that a low heart rate in HFrEF patients in SR improves prognosis and reduces clinical endpoints: for example, a retrospective analysis of the CHARM (Candesartan in HF: Assessment of Reduction in Mortality and Morbidity) program in HFrEF and HFpEF patients, showed a clear correlation between an increased heart rate and endpoints such as New York Heart Association class and hospitalization due to HF.^49^ Similarly, a reduction in heart rate with ivabradine in HF patients with SR has convincingly shown an improvement in prognosis.^48^ In principle, the negatively chronotropic effect of beta‐blockers is also important for AF patients,^50^, ^51^ although there is no clear evidence of the benefit of bradycardic therapy or of an optimal target frequency for these patients. Beta‐blockers such as carvedilol may also help prevent the occurrence of AF in patients with left ventricular dysfunction, as shown in the CAPRICORN (Carvedilol Post‐Infarct Survival Control in Left Ventricular Dysfunction) trial in patients after myocardial infarction.^52^ In a meta‐analysis by Nasr *et al*, which included patients with HF using various beta‐blockers (carvedilol/CAPRICORN^52^/COPERNICUS,^53^ bisoprolol/CIBIS‐I,^54^ metoprolol/MERIT‐HF^43^ and Waagstein *et al*.,^55^ bucindolol/BEST^56^ and nebivolol/SENIORS^45^), beta‐blocker therapy was associated with a significant reduction in the incidence of new AF from 39 to 28 per 1000 patient‐years (relative risk reduction 27%; 95% confidence interval [CI] 14–38, *p* < 0.001).^57^ In the SENIORS (Study of the Effects of Nebivolol Intervention on Outcomes and Rehospitalization in Seniors with Heart Failure) trial, however, nebivolol was not associated with a reduction in new‐onset AF,^45^ possibly due to the high number of older patients with HFpEF.

Despite these beneficial effects, it has not been clearly established whether beta‐blockers have a positive prognostic effect in patients with HFrEF and pre‐existing AF in terms of morbidity and mortality. In a meta‐analysis of 10 randomized controlled trials comparing beta‐blockers with placebo in HF, Kotecha and colleagues analysed the outcome separately in patients with SR and those with AF.^58^ The primary endpoint was all‐cause mortality. Beta‐blocker therapy resulted in a significant reduction in all‐cause mortality in patients with SR (hazard ratio [HR] 0.73, 95% CI 0.67–0.80; *p* < 0.001), but not in patients with AF (HR 0.97, 95% CI 0.83–1.14; *p* = 0.73), with a significant difference for the interaction of the baseline rhythm (*p* = 0.002). A further meta‐analysis of four studies, which included a total of 8680 patients with HFrEF, came to a similar conclusion: beta‐blockers improved mortality in patients with SR (*n* = 7003), whereas no significant benefit was observed in patients with AF (*n* = 1677).^59^ Furthermore, in AF patients, no effect on the second important outcome in HFrEF patients was detectable: a reduced rate of hospitalizations due to HF, in contrast to patients in SR.^59^ Another large meta‐analysis by Kotecha *et al*.^60^ of patients with HFrEF treated with beta‐blockers confirmed these results: a significant improvement in prognosis in patients with SR (*n* = 14 166) but not with AF (*n* = 3034). Finally, a more recent individual patient‐level analysis of 17 312 patients from 11 double‐blind randomized trials demonstrated that for patients in AF with <50% left ventricular ejection fraction (LVEF), beta‐blockers increased LVEF but did not improve prognosis.^61^

These surprising results challenge our understanding of HF pharmacotherapy and the pathophysiology of AF with concomitant HF, but they are not without controversy. An observational nationwide cohort study (39 741 patients with HF and AF) of beta‐blockers showed improved mortality rates in patients with AF and HF.^62^ These results conflict with the previously mentioned study,^61^ which may partly be due to the different design of the studies, as the latter study did not make a clear distinction between HFpEF, HFrEF and HFmrEF patients.^62^ Nevertheless, it may be of clinical importance to consider the differences between patients in SR and AF. A critical difference could be the significance of heart rate: while this should be low in HF and SR, as noted above, this is not so clear in AF. The lowest risk of cardiovascular endpoints seems to be a heart rate in the range of 70 to 110 bpm for AF and HF (the ‘Goldilocks zone’ of AF in HF^63^). It is conceivable that the irregularity of the heartbeats is particularly noticeable at lower heart rates.^64^ This emphasizes the previously underestimated importance of the ‘arrhythmogenicity’ of the heart rate sequence for the development of cardiomyopathy and HF.^31^, ^32^, ^65^ Between 70 and 110 bpm, a certain pseudo‐normalization of the arrhythmic heartbeat sequence may occur, which is possibly prognostically favourable,^66^, ^67^ before tachycardia predominates at a pulse rate >110 bpm and the tachycardia component prevails.^63^, ^68^ In line with this concept, in patients with permanent AF and HF, strict rate control (<70–80 bpm) offered no outcome benefit over lenient control (<110 bpm) and was linked to more adverse effects, as was demonstrated in the RACE II (Rate Control Efficacy in Permanent AF II) trial.^69^ However, it should also be mentioned that strong data regarding the pathophysiology of AF in HF and, in particular, the use of beta‐blockers in AF and HF are still pending.^70^ The studies presented here that cast doubt on the benefits and those that indicate a benefit are difficult to compare due to differences in study design and, for example, the imprecise definition of the groups HFrEF, HFmrEF, and HFpEF. Moreover, most of the trials listed have examined AF in a completely different way than the current standard requires. For example, most studies did not focus on whether AF was paroxysmal, permanent, or newly onset, or if patients with previous AF were in SR during the study. Additionally, it is unclear who was effectively treated with rhythm control and only experienced AF before the trial. All these factors make it difficult to assess the effect of beta‐blockers alone in different AF patients regarding the pharmacotherapy of their HF. New studies are warranted to elucidate this (see the call to action). Since no large, well‐designed studies for patients with HF and AF exist so far, the large studies of patients with HF and SR and their results should currently apply. Therefore, guidelines recommend beta‐blocker use in principle for patients with HF, in particular HFrEF and HFmrEF,^15^ including those with AF.

### Angiotensin‐converting enzyme inhibitors/angiotensin receptor blockers

Angiotensin‐converting enzyme inhibitors are a key component of therapy for HFrEF and with slightly less evidence for HFmrEF.^15^ They were the first class of drugs to show efficacy in HFrEF, both in terms of prognosis and symptoms.^71^, ^72^, ^73^ ARBs have place in HF therapy especially when ACE inhibitors are contraindicated. For example, in the CHARM‐Alternative study, candesartan reduced the number of deaths and hospitalizations in patients who did not receive ACE inhibitors due to previous intolerance.^74^ The evidence that ACE inhibitors and ARBs can help prevent the recurrence of AF in HF patients is relatively solid: an evaluation of the SOLVD (Studies of Left Ventricular Dysfunction) trials showed that enalapril reduces the incidence of AF in patients with left ventricular dysfunction.^75^ A further analysis of the SOLVD trials showed that ACE inhibitors reduce the frequency of hospital admissions for tachycardic AF.^76^ A similar effect has been demonstrated for ARBs in HF patients: in a composite analysis of the CHARM trials, candesartan reduced the incidence of AF in HF patients regardless of ejection fraction.^77^ In the RACE 3 trial, ACE inhibitors or ARBs were part of a targeted therapy for patients with early persistent AF and incipient HF (*n* = 245). Together with mineralocorticoid receptor antagonists (MRAs), statins, and cardiac rehabilitation, SR maintenance was significantly improved in the intervention group compared to the control group (odds ratio 1.765, lower limit of 95% CI 1.021, *p* = 0.042).^78^ ACE inhibitors and ARBs can therefore be regarded as AF‐preventive drugs for patients with HF.^79^ But what is the evidence regarding the efficacy of these drugs in patients with HF and concomitant AF? To our knowledge, there is a lack of secondary analyses from the large prospective studies regarding the effect of ACE inhibitors in the subgroup of HF patients with concomitant AF. Only the ARB candesartan has thus far been shown to significantly reduce the risk of adverse outcomes in patients with AF and HF along the entire spectrum from reduced to preserved systolic function.^80^ Thus, with ACE inhibitors, as with beta‐blockers, there is no clear evidence of benefits in HF patients with AF. However, this therapy is important for these patients. Regarding ACE inhibitors/AT antagonists – like beta‐blockers – new, well‐designed trials are needed to specifically evaluate patients with AF and HF, not just dichotomously, that also break down and categorize AF according to type (paroxysmal vs. permanent) and duration.

### Angiotensin receptor–neprilysin inhibitors

The recommendation to use the angiotensin receptor–neprilysin inhibitor (ARNI) sacubitril/valsartan in patients with HFrEF^15^ who remain symptomatic on ACE inhibitors is based on the results of the PARADIGM‐HF (Prospective comparison of ARNI with ACE Inhibitor to Determine Impact on Global Mortality and morbidity in Heart Failure) study.^81^ Sacubitril/valsartan was shown to be superior to enalapril in reducing hospitalizations for worsening HF, cardiovascular mortality, and all‐cause mortality in patients with ambulatory HFrEF with LVEF <40%. The evidence from this study relates to outpatients with stable HF. In addition, sacubitril/valsartan may also reduce the risk of rehospitalization for HF in hospitalized patients with decompensated HF.^82^, ^83^

Of note, the subgroup of patients with HFrEF and previous AF was explicitly investigated in the PARADIGM‐HF study. Among 8399 trial participants, 3091 had AF. In this subgroup, the use of sacubitril/valsartan resulted in a similar risk reduction with regard to the primary endpoint (composite of death from cardiovascular causes or hospitalization for HF) in both the overall cohort and patients in SR.^81^ Also, given that the guideline recommendation is mainly based on this one large study (level of evidence IB),^15^, ^81^ there is certainly sufficient evidence to also treat patients with AF and HFrEF who are symptomatic on ACE inhibitors with sacubitril/valsartan to reduce morbidity and mortality. However, a stronger effect of sacubitril/valsartan to prevent AF in HF patients versus ACE inhibitors and ARBs has not been clearly demonstrated. Mohammad *et al*.^84^ performed a recent retrospective analysis of 11 studies (11 458 patients on sacubitril/valsartan, 10 128 patients on ACE inhibitors/ARBs) that showed that although sacubitril/valsartan improved mortality versus ACE inhibitors/ARBs, it did not reduce the risk of developing AF. A comparison of sacubitril/valsartan versus placebo was understandably not performed, and, therefore, it has not been clearly shown that sacubitril/valsartan reduces the occurrence of AF. Interestingly, a secondary analysis of patients with AF (*n* = 3449) in PARADIGM‐HF (combined with patients from the ATMOSPHERE [Aliskiren Trial to Minimize Outcomes in Patients with Heart Failure] trial^85^) emphasized the further discussed principle that a low heart rate in HF and concomitant AF is rather detrimental: heart rates ≤72 bpm in these patients were associated with higher risk of HF‐associated death.^86^

### Mineralocorticoid receptor antagonists

Aldosterone as a key target molecule of the upregulated renin–angiotensin–aldosterone system in HF is responsible for many deleterious effects in HF, such as increased cardiac (and also renal) fibrosis, inflammation, salt and water retention, by binding to its target receptor.^87^ The MRAs spironolactone or eplerenone, together with ACE inhibitors and beta‐blockers, are a fundamental component of HFrEF and HFmrEF therapy and are among the four pillars of HFrEF treatment.^15^, ^40^ The successive application after ACE inhibitors and beta‐blockers may be due to historical reasons related to later large pivotal studies (RALES [Randomized Aldactone Evaluation Study], 1999^88^; EMPHASIS‐HF [Eplerenone in Mild Patients Hospitalization and Survival Study in Heart Failure], 2011^89^) rather than lower efficacy in HF. Eplerenone, for example, has indeed this effect in HF patients: in a secondary analysis of the EMPHASIS‐HF trial, Swedberg and co‐workers showed that new‐onset AF was significantly reduced by eplerenone in HF patients with mild symptoms.^90^ However, it was shown in the same study that the effective reduction in the primary endpoint (cardiovascular death or hospital admission for worsening HF) in patients with AF was similar to that in patients without AF (HR 0.60, 95% CI 0.46–0.79 vs. HR 0.70, 95% CI 0.57–0.85; *p* for interaction = 0.41).^90^ To our knowledge, the proportion of patients enrolled in the RALES trial (spironolactone vs. placebo) with AF is not reported.^88^

The non‐steroidal MRA finerenone represents a new treatment option for cardiorenal patients. In patients with HFmrEF and HFpEF finerenone resulted in a significantly lower rate of a composite of total worsening HF events and death from cardiovascular causes than placebo.^91^ Several ongoing trials are evaluating finerenone in a wider range of patients, including patients with HFrEF (FINALITY‐HF).^92^ This could lead to a new and promising data set in the comorbidity of HFrEF and AF that we are considering.

### Sodium–glucose co‐transporter 2 inhibitors

Originally marketed for the treatment of diabetes mellitus, SGLT2 inhibitors have revolutionized the treatment of HF. They cause glicosuria by inhibiting SGLT2 in the proximal tubule of the kidney and thereby lower blood glucose levels. Potential effects on the heart, range from increased cardiac ketone turnover and decreased cardiac fibrosis to effects on inflammation and oxidative stress.^93^ The SGLT2 inhibitors dapagliflozin and empagliflozin are pillars of therapy for HFrEF, based on the important DAPA‐HF (Dapagliflozin And Prevention of Adverse‐outcomes in HF) trial^94^, ^95^ and the EMPEROR‐Reduced (Empagliflozin Outcome Trial in Patients with Chronic HF and a Reduced Ejection Fraction) trial.^96^ In one sense, this class of medication stands out: firstly, we have a highly effective class of drugs for the treatment of cardiorenal syndrome.^97^, ^98^ Moreover, in contrast to beta‐blockers and, to some extent, ACE inhibitors and ARNI, SGLT2 inhibitors can be used with good evidence in HF across the entire spectrum of LVEF, including HFpEF.^40^

The effect of dapagliflozin in HFrEF patients with AF was similarly beneficial. In a secondary analysis of the DAPA‐HF trial, out of 4744 randomized patients, 1910 (40.3%) had AF. Compared to placebo, dapagliflozin reduced the combined endpoint of cardiovascular death and worsening HF equally in patients with and without AF (HR 0.75, 95% CI 0.62–0.92, and HR 0.74, 95% CI 0.62–0.88, respectively; *p* for interaction = 0.88).^99^ These data clearly emphasize the importance of SGLT2 inhibitors in patients with HF and AF.

In a meta‐analysis that included various studies of heart and kidney failure patients (the EMPA‐REG OUTCOME trial, CANVAS, CANVAS‐R, the DECLARE‐TIMI 58 trial, CREDENCE, DAPA‐HF, VERTIS‐CV and DAPA‐CKD), Okunrintemi and colleagues showed a significantly lower incidence of AF in individuals taking SGLT2 inhibitors versus placebo (relative risk 0.79, 95% CI 0.67–0.93).^100^ It can therefore be assumed that SGLT2 inhibitors have a certain preventive effect regarding the first occurrence of AF in HF patients.

### Ferric carboxymaltose

Iron deficiency (defined as transferrin saturation <20% or serum ferritin <100 μg/L) is a very common comorbidity in patients with HF that is associated with a worsened outcome and increased symptoms.^101^, ^102^ Intravenous ferric carboxymaltose has been shown in various studies to be an effective therapy for improving the symptoms of HF.^103^, ^104^ Based on meta‐analyses^105^, ^106^ showing that intravenous iron infusion in patients with HF reduces the composite risk of first hospitalization for HF and cardiovascular mortality as well as the risks of first and recurrent hospitalizations for HF, with no effect on all‐cause mortality or cardiovascular mortality alone, the focussed update of the ESC guidelines for the diagnosis and treatment of acute and chronic HF recommends intravenous iron supplementation in patients with HF and iron deficiency (Class I Level A to improve symptoms, Class IIa level A to prevent hospitalization for HF).^40^ To the best of our knowledge, neither the large foundational studies nor the meta‐analyses have analysed the efficacy of this therapy in patients with concomitant HF, iron deficiency, and AF. However, it seems advisable to investigate this, as AF is often associated with iron deficiency.^107^ The fact that a large proportion of those affected are treated with anticoagulants and therefore have an increased risk of bleeding emphasizes the importance of the subgroup of patients with iron deficiency, AF, and HF. In a recent study by Mentz and co‐workers in 3065 outpatients who had HFrEF and iron deficiency, the administration of ferric carboxymaltose did not significantly reduce the hierarchical composite endpoint of death and hospitalization for HF or improve the 6‐min walking distance.^108^ In this study, the subgroup of patients with AF was analysed selectively and, as in the overall cohort, there was no significant reduction in the HR.^108^

### Vericiguat

Vericiguat is a novel oral soluble guanylate cyclase stimulator that enhances the cyclic guanosine monophosphate pathway by directly stimulating the enzyme through a binding site independent of the effects of nitric oxide.^109^ In a large study of 5050 high‐risk HFrEF patients with recent hospitalization or intravenous diuretic therapy (VICTORIA [Vericiguat Global Study in Subjects with Heart Failure with Reduced Ejection Fraction] trial), vericiguat significantly reduced the endpoint incidence of death from cardiovascular causes or hospitalization for HF.^110^ In a secondary analysis of this trial, Ponikowski and co‐workers investigated the relationship between baseline and new‐onset AF and outcome.^111^ Patients were classified into three groups: no known AF (*n* = 2661, 53%), history of AF alone (*n* = 992, 20%), and AF on randomization electrocardiogram (*n* = 1357, 27%). Neither type of AF affected the beneficial effect of vericiguat. Development of AF post‐randomization was associated with an increase in both cardiovascular death and HF hospitalization. Of note, the rate of new‐onset AF in HF patients was not affected by vericiguat.^111^

### Heart failure with improved ejection fraction

Our article focuses on patients with HFrEF and AF, but an important question is how to treat the group of patients with HF with improved ejection fraction (HFimpEF).^112^ Two therapeutic approaches in patients with AF could lead to an improvement in ejection fraction: antiarrhythmic therapy for AF and guideline‐directed medical therapy for HF. According to current knowledge, if left ventricular pump function is restored after arrhythmia treatment, the prognosis for survival is good.^113^ A novel report by Domínguez‐Rodríguez and coworkers showed that in the setting of AiCM with improved LVEF, maintaining renin–angiotensin system inhibitors and beta‐blockers was associated with a significantly lower incidence of relapse.^114^ However, this study had some weaknesses, and AiCM patients were mixed with dilated cardiomyopathy patients due to the time to diagnosis. Moreover, it is not clear whether patients experienced worsening ejection fraction without arrhythmia relapse, which would suggest a ‘true’ cardiomyopathy in this collective. Therefore, this complex problem also needs to be further investigated by prospective trials. In any case, present knowledge advocates for great caution in discontinuing HF medication and close clinical monitoring thereafter.^115^ In this regard, one study showed that even years after AiCM and normalization of left ventricular systolic dysfunction on magnetic resonance imaging, dilatation and ultrastructural myocardial lesions can be detected.^116^

## Conclusions and a call to action

**Figure 2:**
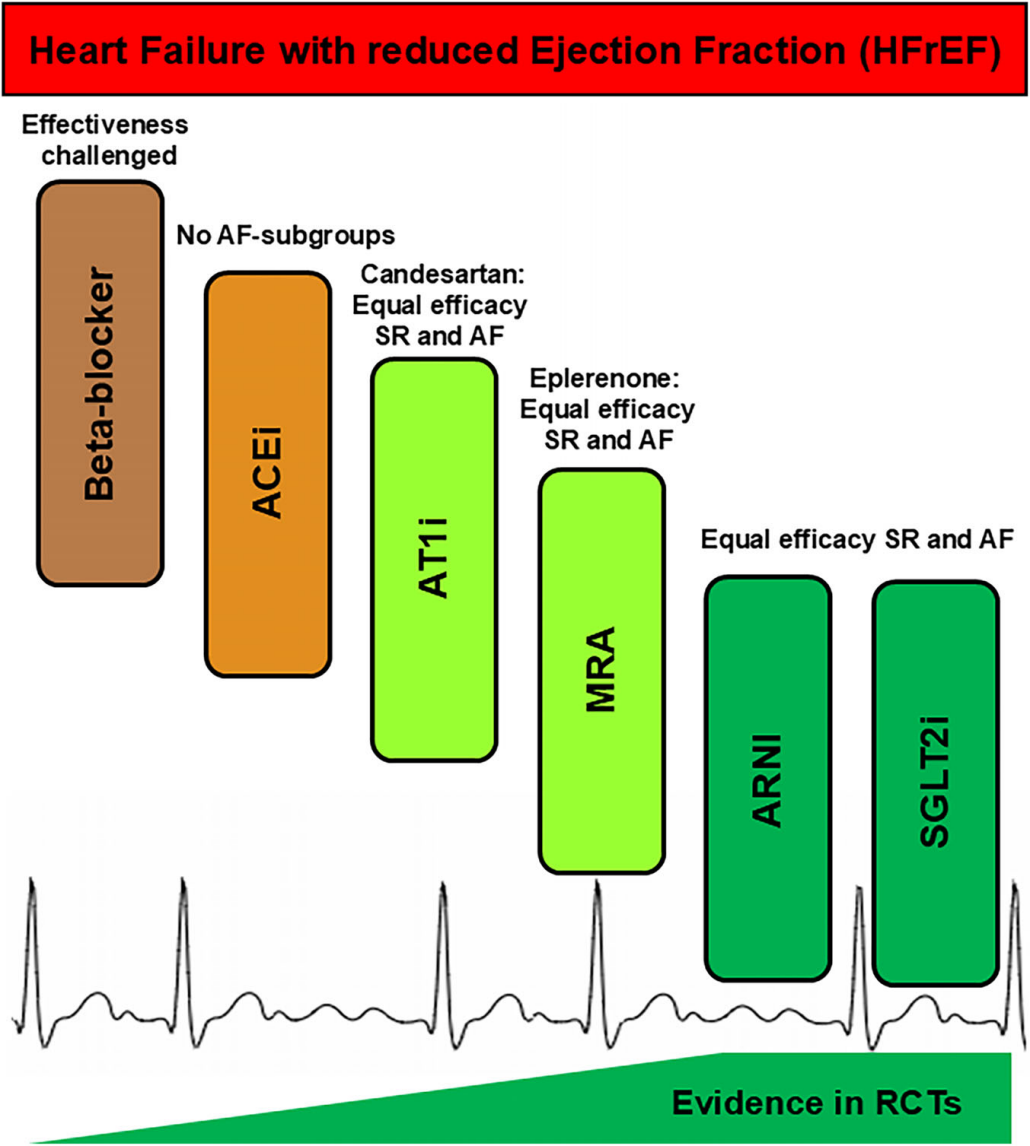
Current evidence for class I‐recommended, guideline‐directed medical therapy in heart failure with reduced ejection fraction (HFrEF) patients with concomitant atrial fibrillation (AF). There are significant differences among long‐established HFrEF therapies, not necessarily in terms of efficacy but in the level of evidence supporting their effectiveness based on the results of randomized controlled trials (RCTs). Overall, the efficacy of long‐established HFrEF therapies, such as beta‐blockers, angiotensin‐converting enzyme inhibitors (ACEi), and to some extent angiotensin receptor type 1 antagonists (AT1i), angiotensin receptor blockers and mineralocorticoid receptor antagonists (MRAs), has been less extensively studied compared to more recently approved treatments like sodium–glucose co‐transporter 2 inhibitors (SGLT2i) or angiotensin receptor–neprilysin inhibitors (ARNI) in relation to AF. The efficacy of beta‐blockers in patients with HFrEF and AF remains a topic of debate, as their benefit in this population is uncertain. For other established therapies, such as ACEi, AT1i, and angiotensin receptor blockers, evidence remains heterogeneous, and available data on AF are limited to certain agents within these drug classes. However, there is currently no evidence to support the discontinuation of these well‐established therapies in patients with AF and HFrEF, underscoring the need for further research and greater awareness. SR, sinus rhythm.

We have reviewed the evidence for the effectiveness of standardized HF pharmacotherapy in patients with concomitant AF (*Table* *1*). There are major differences, perhaps not so much in terms of efficacy, but at least in terms of evidence of efficacy (*Figure* *2*). In general, the efficacy of well‐established HF therapies such as beta‐blockers in relation to AF has been less extensively studied compared to more recently approved treatments like SGLT2 inhibitors or vericiguat. We are not suggesting that well‐established HF therapies such as beta‐blockers should be discontinued in patients with AF and HFrEF. Rather, one of our goals is to highlight the significant gaps of knowledge in both fundamental and clinical research and clinical practice: in recent years, organ‐focused therapy of the heart has taken a back seat because the enormous importance of comorbidities in HF has been increasingly recognized.^14^, ^15^, ^40^, ^117^, ^118^, ^119^ Cardiorenal syndrome has been consistently identified and scientifically addressed, which has ultimately led to a tailored therapy for cardiorenal syndrome with the use of SGLT2 inhibitors.^97^, ^98^ Cardiologists have also acknowledged the special needs of diabetic patients and addressed them with their own clinical guidelines based on scientific findings.^120^, ^121^

**Table 1:** Evidence of heart failure drugs in patients with heart failure with reduced ejection fraction in sinus rhythm versus atrial fibrillation

Drug class	Benefit in HFrEF (sinus rhythm)	Benefit in HFrEF (AF)	Prevention of new‐onset AF	Key references	Key studies
Beta‐blockers	Strong (multiple RCTs e.g. MERIT‐HF, CIBIS‐II, COPERNICUS)	Limited benefit in AF; neutral effect on mortality (e.g. Cleland, Kotecha)	Yes (e.g. CAPRICORN, Nasr meta‐analysis)	MERIT Study Group,^43^ McMurray,^52^ Packer,^53^ Kotecha,^58^ Cleland ^61^	MERIT‐HF, CIBIS‐II, COPERNICUS, CAPRICORN
ACE inhibitors/ARBs	Strong (e.g. SOLVD, CONSENSUS, CHARM)	Mixed; CHARM‐AF sub‐analysis with modest benefit	Yes (SOLVD, CHARM, RACE 3)	Vermes,^75^ Rienstra 3,^78^ Olsson^80^	SOLVD, CONSENSUS, CHARM
ARNI (sacubitril/valsartan)	Strong (PARADIGM‐HF)	Benefit in subgroup with AF (PARADIGM‐HF, ATMOSPHERE)	Unclear (meta‐analyses inconsistent)	MCMurray,^81^ Krum ^85^	PARADIGM‐HF, ATMOSPHERE
MRAs	Strong (RALES, EMPHASIS‐HF)	Positive in AF subgroup (e.g. EMPHASIS‐HF)	Yes (e.g. EMPHASIS‐HF secondary analyses)	Pitt,^88^ Zannad ^89^	RALES, EMPHASIS‐HF
SGLT2 inhibitors	Strong (DAPA‐HF, EMPEROR‐Reduced, DELIVER)	Consistent benefit in AF and sinus rhythm (e.g. DAPA‐HF, DELIVER)	Yes (Okunrintemi meta‐analysis, consistent across studies)	McMurray,^94^ Packer,^96^ Okunrintemi^100^	DAPA‐HF, EMPEROR‐Reduced, DELIVER
Ferric carboxymaltose	Improves symptoms, reduces HF hospitalizations (FAIR‐HF, CONFIRM‐HF)	Uncertain; subgroup analysis not conclusive	Unclear (no specific prevention data)	Anker,^103^ Ponikowski,^104^ Mentz^108^	FAIR‐HF, CONFIRM‐HF, HEART‐FID
Vericiguat	Moderate (VICTORIA trial)	Positive in AF subgroup (VICTORIA AF analysis)	No effect on new‐onset AF	Armstrong^110^ Ponikowski^111^	VICTORIA, Ponikowski

ACE, angiotensin‐converting enzyme; AF, atrial fibrillation; ARB, angiotensin receptor blocker; ARNI, angiotensin receptor–neprilysin inhibitor; HFrEF, heart failure with reduced ejection fraction; MRA, mineralocorticoid receptor antagonist; RCT, randomized controlled trial; SGLT2, sodium–glucose co‐transporter 2.

AF, atrial fibrillation; AiCM, arrhythmia‐induced cardiomyopathy; CRT, cardiac resynchronization therapy; HF, heart failure.

But what can be done to improve the treatment of HFrEF with concomitant AF? Greater precision in therapy could be gained by better appreciation of the different pathophysiologies and disease progression in AF and HF, the different phases and duration of remissions, and the importance of heart rate. Among other aspects, the new guidelines also emphasize the substantial contribution of comorbidities,^51^ as type 2 diabetes mellitus and renal failure. New studies are needed to examine patients with concomitant AF and HF as close as possible specifically for the status of AF. It is probably not enough to conduct further HF studies in which patients with AF are one of many subgroups since those type of analyses are underpowered to assess outcomes. We need to answer more and direct questions about this group, such as what type of AF is present. For example, it can be different whether a patient is in SR during the trial with only a history of AF, or whether there were just a few episodes of paroxysmal AF, or if a patient has long‐standing persistent or permanent AF. In addition, the importance of heart rate control and, even more critical, the significance of rhythm control in addition to HF therapy need further clarification. There is very little information on how or even whether rhythm or rate control was achieved in the large subgroup of patients with AF in most of the large HF trials. Data from the GALACTIC‐HF trial, for example, suggest that the use of digoxin for rate control in patients with AF can attenuate the effect of HF therapy with omecamtiv mecarbil.^122^ In addition, patients with different stages of AF are probably not easily comparable. Moreover, successful rhythm control can influence the prognosis of patients in HF trials. Another open question is related to patients treated with cardiac resynchronization therapy (CRT)^86^ for left bundle branch block and HFrEF. In pharmacological trials, this subgroup is often listed alongside the AF subgroup. But is there an intersection of patients who have AF, HF, and CRT? Here, the issue of rate control may play a much more important role in enabling a sufficient pacing rate and thus effective CRT therapy. In patients for whom all conventional rhythm therapy options have failed, atrioventricular ablation and a CRT system could also be a last‐resort treatment option.^123^ These patients would be particularly interesting to study, since they have regular ventricular rate despite presence of AF.

The underlying cause of HFrEF should also be dissected in future trials. The sole classification of ischaemic versus non‐ischaemic cardiomyopathy as the main cause of HFrEF can hardly reflect our new knowledge about cardiomyopathies. What is the proportion of patients with tachymyopathy in rapid AF? What is the proportion of patients with AiCM,^31^, ^32^ where the principle of rhythm control is possibly even more important than optimal HF therapy, also according to current guidelines? The available studies differentiate little or not at all between those with AiCM and those with AF and HF, although this differentiation has important clinical implications.

Therefore, we need to fill gaps in evidence to better understand our patients with AF and HFrEF and recognize their specific interaction patterns, underlying mechanisms, critical issues, and unmet clinical needs. Our patients are likely to benefit significantly when cardiologists collaborate with specialists from other fields such as diabetologists, nephrologists, oncologists, and others, to address complex issues at the intersection of these specialties in the sense of systems cardiology. To better understand and treat these two comorbidities, however, we may need a lively collaboration within our own disciplines of cardiology, namely between electrophysiologists and HF specialists, which still act as separate disciplines worldwide. Just as HF extends beyond being a mere left ventricular dysfunction, AF transcends being solely an atrial condition. Experts in arrhythmology should consider AF within the broader context of HF, while HF specialists must similarly evaluate AF in HF patients. Often, the critical boundary that remains overlooked lies at the level of the atrioventricular valve, which is crucial for understanding patient's condition. Bridging this gap is essential to comprehensively understanding and treating these interconnected diseases (*Box* *1*). Let's take action!

**Box 1.** Proposed directions for future research in patients with atrial fibrillation and heart failure

1. Stratify AF in HF trials

- Distinguish paroxysmal, persistent, and permanent AF.
- Compare outcomes based on AF timing (pre‐existing vs. new‐onset).
- Avoid treating AF as a homogeneous subgroup in HF studies.

2. Rhythm and rate control: a critical focus

- Study the impact of rhythm control on HF prognosis.
- Evaluate heart rate control strategies in different AF profiles.
- Investigate drug interactions (e.g. digoxin and omecamtiv mecarbil).

3. Comprehensive profiling of HF aetiology

- Move beyond the ischaemic versus non‐ischaemic dichotomy.
- Focus on tachycardia‐induced cardiomyopathy and AiCM.
- Align therapeutic strategies with underlying pathophysiology and disease stage.

4. Re‐assess CRT in the context of AF

- Evaluate the role of rate control in CRT effectiveness.
- Study outcomes in patients with concomitant AF, HF, and CRT.
- Identify those who may benefit from atrioventricular node ablation combined with CRT.

5. Foster multidisciplinary collaboration

- Strengthen collaboration between electrophysiologists and HF specialists.
- Integrate care involving nephrologists, diabetologists, and oncologists.
- Consider the atrioventricular valve as a functional interface, not a barrier.

6. AF‐HF dedicated clinical trials

- Design clinical trials specifically for the AF‐HF population.
- Systematically document AF type, rhythm control status, and treatment response.
- Establish a precision medicine framework tailored to this comorbidity.

### Funding

Prof. Sossalla is funded by the Deutsche Forschungsgemeinschaft (DFG) through projects 471241922, 549060740, the CRC 1213 project B10N, the F. Thyssen Foundation (Az 10.19.2.026MN), and the Excellence Cluster Cardio Pulmonary Institute (CPI). Prof. Böhm is supported by the Deutsche Forschungsgemeinschaft (German Research Foundation; TTR 219, project number 322900939). Prof. Dobrev is supported by the Deutsche Forschungsgemeinschaft (Research Training Group 2989, project 517043330), National Institutes of Health (R01HL131517, R01HL136389, R01HL163277, R01HL160992, R01HL165704, R01HL164838, and R01HL176651), and European Union (large‐scale integrative project MAESTRIA, No. 965286; DD). M.C. Petrie: Research funding – Boehringer Ingelheim, Roche, SQ Innovations, AstraZeneca, Novartis, Novo Nordisk, Medtronic, Boston Scientific, Pharmacosmos.

**Conflicts of interest**: M.L.: speakers/consulting honoraria from AstraZeneca, Boehringer Ingelheim, Novartis, Vifor, Daiichi Sankyo, Bristol Myers‐Squibb, Pfizer. G.A.: consulting and speaker fees from Amarin, CLS, Menarini, Novartis, Novo Nordisk. A.B.G.: personal fees from Abbott, Amgen, AstraZeneca, Bayer, Boehringer Ingelheim, Cytokinetics, Medtronic, Novartis, Servier and Vifor. P.F.: Founder and CEO of Pharmahungary Group, a group of R&D companies. CEO of Pharmahungary Group, a group of R&D companies. E.L.G.: speaker honoraria or consultancy fees from AstraZeneca, Bayer, Boehringer Ingelheim, Bristol Myers Squibb, Pfizer, Novo Nordisk, Lundbeck Pharma; investigator in clinical trials sponsored by AstraZeneca, Viatris or Bayer and has received an unrestricted research grant from Boehringer Ingelheim. Roland Koinenberg (R. K.): R. K.: Speaker fees from Amarin, Amgen, Daiichi Sankyo, Novartis and Pfizer; advisory board and consultancy fees from Amarin, Bristol Myers Squibb, Daiichi Sankyo and Novartis; research funding from Daiichi Sankyo. J.M.: speaker honoraria or consultancy fees from Amarin, AstraZeneca, Bayer Healthcare, Boehringer Ingelheim, Daiichi Sankyo, Janssen, Merck Sharp & Dohme. W.P.: research funding and consulting fees from AstraZeneca. M.C.P.: consultancy and trial committees – Abbott, Abbvie, Akero, Applied Therapeutics, Amgen, AnaCardio, Biosensors, Boehringer Ingelheim, Corteria, Novartis, AstraZeneca, Novo Nordisk, Abbvie, Bayer, Horizon Therapeutics, Foundry, Takeda, Cardiorentis, Pharmacosmos, Siemens, Eli Lilly, Vifor, New Amsterdam, Moderna, Teikoku, LIB Therapeutics, 3R Lifesciences, Reprieve, FIRE 1, Corvia, Regeneron. A. G. S: has received speaker honoraria and/or consulting fee from Merck/Schering‐Plough, BMS, UCB, Pfizer/Wyeth, Novartis, Sanofi, Women's College Hospital, Toronto, Canada, and Rheumatology Association, Finland. M.S.: consultant for Novartis, Abbott, Merck, Merck Sharp and Dohme, Vifor, AstraZeneca, Cardurion, Novo Nordisk, Bayer, and Boehringer Ingelheim. P.S.: grants and personal fees from Boehringer Ingelheim, Bayer and AstraZeneca. M.M.: consulting fees from AstraZeneca, Bayer, Boehringer Ingelheim, Novo Nordisk, Roche Diagnostics; speaker bureau from Boehringer Ingelheim and Zoll therapeutics. D.D.: speaker honoraria or consultancy fees from AbbVie Deutschland, Daiichi Sankyo and Bayer. S.S.: speakers/consulting honoraria from Astra Zeneca, Novartis, Berlin‐Chemie, Daiichi Sankyo, Bristol Myers Squibb, Pfizer, Boehringer Ingelheim, and Lilly. All other authors have nothing to disclose.
